# Characterisation of the sarcomeric myosin heavy chain multigene family in the laboratory guinea pig

**DOI:** 10.1186/1471-2199-11-52

**Published:** 2010-06-29

**Authors:** Daniel P Tonge, Simon W Jones, Ronald G Bardsley, Tim Parr

**Affiliations:** 1 Nutritional Sciences School of Biosciences Sutton Bonington Campus University of Nottingham LE12 5RD, UK; 2 Respiratory and Inflammation Research AstraZeneca, Alderley Park, Macclesfield, Cheshire, SK10 4TG, UK

## Abstract

**Background:**

Several chronic conditions leading to skeletal muscle dysfunction are known to be associated with changes in the expression of myosin heavy chain (MHC) isoforms at both the mRNA and protein level. Many of these conditions are modelled, pre-clinically, in the guinea pig due to similar disease onset and progression to the human condition, and their generally well-characterised anatomy. MHC composition is amenable to determination by protein and mRNA based methodologies, the latter quantifying the expression of MHC isoform-specific gene transcripts allowing the detection of earlier, and more subtle changes. As such, the MHC mRNAs, and specific oligonucleotide primers of all common laboratory species have been available for some time. However, due to incomplete genomic annotation, assessment of guinea pig MHC mRNA expression has not been previously possible, precluding the full characterisation of early changes in skeletal muscle in response to disease and disease modulation.

The purpose of this study was to characterise the multigenic structure of the sarcomeric MHC family in the guinea pig, and to design and validate specific oligonucleotide primers to enable the assessment of the predominant adult-muscle associated MHC mRNAs in relevant disease models.

**Results:**

Using a combination of ligase-mediated rapid amplification of 5' and 3' cDNA ends (RACE) and bioinformatics, mRNAs to the four main skeletal-muscle isoforms of MHC were determined. Specific oligonucleotide primers were designed, and following verification of their specificity, found to successfully determine the expression of each MHC mRNA independently.

**Conclusions:**

Because of their utilisation in the *in vivo *modelling of disease, there is a requirement to develop molecular methods that accurately differentiate the different MHC mRNAs in the guinea pig to enable rapid profiling of muscle composition in appropriate disease models. The methods developed here are suitable for the characterisation of muscle MHC expression at the molecular level from animal tissue samples and biopsy material. The publication of these specific oligonucleotide primers for the guinea pig MHC variants will enable researchers to rapidly and accurately quantify acute changes in MHC mRNA expression in either developmental or in guinea pig disease models where a marker of altered skeletal muscle function is required.

## Background

Skeletal muscle tissue is able to adapt to various stimuli including neuronal, hormonal, mechanical and nutritional signals [1]. Termed "plasticity", this process of adaption is made possible, in part, by the existence of multiple isoforms of myosin heavy chain (MHC), which comprise a family of molecular motors able to modulate the speed of skeletal muscle contraction [1]. The sarcomeric MHC family consists of at least eleven isoforms, eight of which are encoded by distinct genes located in two multigenic regions on two separate chromosomes [2]. Six genes are encoded by a 300 - 600 Kb segment on human and mouse chromosomes 17 and 11 respectively, in a cluster arrangement in the order MyH3/MyH2, MyH1/MyH 4, MyH 8/MyH13. The MyH2, MyH1 and MyH4 genes encode the protein isoforms commonly termed MHC IIA, IIX and IIB. The slow-type MHC skeletal muscle (Type 1 or MyH7β) and cardiac (MyHα) isoforms are located independently from the other striated muscle associated isoforms on chromosome 14 in both species [2]. The remaining three MyH genes, named MyH14, MyH15 and MyH16 have only recently been described [3] and are found on human chromosomes 20, 3 and 7 respectively. Of the eleven sarcomeric isoform genes of MHC, four are known to be expressed in adult skeletal muscle: one "slow-twitch" (Type I) muscle-associated MHC isoform and three "fast-twitch" (Types IIA, IIX and IIB) muscle-associated isoforms. The expression of MHC IIB protein is species-specific and has been described in marsupials and various laboratory strains [4], but is absent in human [5], primate, bovine, canine, and feline locomotor muscles [6].

Several chronic pathological conditions leading to skeletal muscle dysfunction are known to be associated with changes in the relative proportions of MHC of various skeletal muscles. Age-related sarcopenia has been characterised by a decrease in MHC IIB and IIX protein leading to an overall transition from fast to slower muscle fibre characteristics [7]. Osteoarthritis has been associated with the loss of MHC IIX and IIA fibres [8] in the quadriceps muscle, whilst chronic heart failure (CHF) has been associated with a significant decrease in the cardiac complement of MHC Iα [9]. Changes in skeletal muscle properties have only recently been described in chronic obstructive pulmonary disease (COPD) patients, the diaphragms of which were found to have reduced MHC IIX protein [10].

In order to understand the complex molecular events involved in the initiation and progression of such disorders, and in the development of pharmacological agents that modulate disease, animal models that attempt to mimic human pathology are often utilised. The laboratory guinea pig (*Cavia porcellus*) is used for *in vivo *modelling of chronic disorders including heart failure [11], osteoarthritis [12] and COPD [13].

In attempting to monitor changes in MHC expression as markers for skeletal muscle contractile function, a number of methods exist including traditional histochemical studies [14], electrophoretic separation of MHC [15,16], and the use of MHC-specific anti-sera [7]. Although well characterised, these methodologies focus on the determination of MHC protein and are therefore restricted to the assessment of changes post-translation. More recently, molecular-based methodologies have focused on the quantification of MHC isoform-specific mRNA, allowing the detection of global changes in MHC gene expression that may precede changes in MHC protein, although translational control mechanisms undoubtedly exist. Thus far, MHC gene expression has been quantified in man [17], the rat [18] and various other laboratory species. However, despite their use as an *in vivo *model of human disease associated with muscle dysfunction, due to incomplete genomic annotation, assessment of guinea pig MHC mRNA expression has not been previously possible. Moreover, it appears that attempts to resolve guinea pig MHC isoform-specific proteins by electrophoretic separation have also proved unsuccessful thus precluding the characterisation of changes in skeletal muscle in response to disease and disease modulation.

Because of their utilisation in the *in vivo *modelling of disease, there is a requirement to develop molecular methods that accurately differentiate the different MHC mRNAs in the guinea pig to enable rapid profiling of muscle composition in appropriate animal disease models. Moreover, given that little is known about this species with respect to skeletal muscle MHC expression, the determination of MHC mRNAs is important both in advancing our knowledge of the guinea pig and in the comparison of MHC expression profiles with other rodent species.

The purpose of this study was therefore to characterise the multigenic structure of the sarcomeric MHC gene family in the *Cavia porcellus*, and to design and validate specific oligonucleotide primers to enable the assessment of the four sarcomeric MHC mRNAs expressed in adult skeletal muscle.

## Results and Discussion

### Study Overview

Through a combination of RNA ligase-mediated rapid amplification of 5' and 3' cDNA ends (RACE) and bioinformatics, we aimed to characterise the myosin heavy chain (MHC) multigene family predominantly expressed in guinea pig skeletal muscle (*Cavia porcellus*), and subsequently design specific oligonucleotide primer pairs for the quantification of gene expression of the four major skeletal muscle-associated MHC isoforms.

### Generation of a Short Fragment of Guinea Pig cDNA

In the first instance, a short fragment (626 bp) of guinea pig sequence was generated from quadriceps cDNA using primer pair GP_626 bp (Table 1) targeting a region between exons 5 and 10 that is completely conserved across the three "fast" isoforms of myosin heavy chain in related species (*Rattus norvegicus *and *Mus musculus*) using endpoint PCR. Following cloning and sequencing, this novel sequence was used to interrogate the partially annotated *Cavia porcellus *gDNA database using BLAT (Ensemble release 56) to include sequences from *Rattus norvegicus*, *Mus musculus and Cavia porcellus*. BLAT analysis revealed this sequence to be located at the 5' of gene MyH2 in both *Rattus norvegicus *and *Mus musculus*.

**Table 1:** Comprehensive list of oligonucleotide primers used for RACE and quantitative PCR

Primer Name	Forward (5' - 3')	Reverse (5' - 3')	Tm (°C)	Expected Product (bp)
GP_626 bp	TGTCATCCAGTACTTTGCAACAA	TGAGCAGGTCAGCAGAGTTC	60	626
Reverse_GSP_2	-	CAGGCTGCGTAACGCTCTTTGAGGTTGT	68	-
Reverse_Nested_GSP_2	-	CTCGTGCAGGTGGGTCATCATGG	68	-
Forward_GSP_2	CATGATGGCCGAGGAGCTGAAGAAG	-	68	-
Forward_Nested_GSP_2	AAGGGCGGCAAGAAGCAGATCCAGAAG	-	68	-
MyH7Term3'	AAGTATCGCAAGGCTCAA	CCTTTCCTTAATTCCAAGC	55	129
MyH13'UTR	TTCATCCAAATGCAGGAAAG	TCTTTATCTCAAAAGTCATAAATACAA	55	90
MyH23'UTR	TGTGGAATGACCAGAGCAAG	CCTTTGCAATAGGGTAGGACA	55	85
MyH43'UTR	TCCATCTACTGCTGCAACG	ACTCTGCAGATTTTATTTCCTTG	55	93

### Determining the Genomic Location of Other Members of the Myosin Heavy Chain gene Family

**Figure 1:**
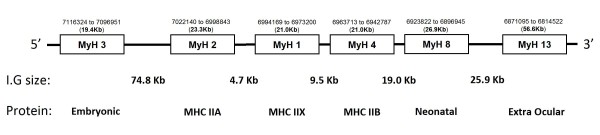
**Spatial arrangement of the multigenic myosin heavy chain (MHC) family in the laboratory guinea pig (Cavia porcellus) as determined through the retrieval of MHC isoform specific genes (MyH) from various species using multiple BLAST and subsequent alignment (ClustalW2)**. I.G - Intergenic region size (Kb); Protein - protein product of each gene.

In all other species described, the myosin heavy chain family of genes is located on a single chromosome and arranged in a head to tail fashion in the order MyH3/MyH2, MyH1/MyH 4 and MyH8/MyH13 with the exception of the cardiac (MyH7α) and slow skeletal muscle isoforms (MyH7β) which are located on a distant chromosome [2]. Using this knowledge, and the previously determined location of the MyH2 gene as a starting point, we were able to advance along the genomic sequence data (Ensemble release 56), aligning individual exons from both *Rattus norvegicus *and *Mus musculus *MyH1, MyH2, MyH3, MyH4, MyH8 and MyH13 cDNAs (Table 2) to the guinea pig gDNA database, thus spatially mapping out the arrangement of genes in the guinea pig (Figure 1). During the course of this study, a full length cDNA pertaining to *Cavia porcellus *MyH7 was annotated in Ensemble release 56. We therefore focused on characterising the remaining fast MHC genes.

**Table 2:** List of gene names, sequence attributes and accession numbers

Species	Gene Name	Accession Number	Comments
*Mus musculus*	MyH1	NM_030679.1	Full Length CDS
	MyH2	NM_001039545.2	Full Length CDS
	MyH3	NM_001099635.1	Full Length CDS
	MyH4	NM_010855.2	Full Length CDS
	MyH8	NM_177369.3	Full Length CDS
	MyH13	NM_001081250.1	Full Length CDS

*Rattus norvegicus*	MyH1	NM_001135158.1	Full Length CDS
	MyH2	NM_001135157.1	Full Length CDS
	MyH3	NM_012604.1	Full Length CDS
	MyH4	NM_019325.1	Full Length CDS
	MyH8	XM_001080186.1	Full Length CDS
	MyH13	XM_001078857.1	Full Length CDS

*Cavia porcellus*	MyH1	GU288593	5' CDS + UTR
	MyH1	GU288596	3' CDS + UTR
	MyH2	GU288594	5' CDS + UTR
	MyH2	GU288597	3' CDS + UTR
	MyH4	GU288595	5' CDS + UTR
	MyH4	GU288598	3' CDS + UTR
	MyH7β	ENSCPOG00000004208	Full Length CDS

### Determination of the Gene Boundaries of MyH1, 2 and 4 by 5' and 3' Race

To accurately determine the boundaries of each of the adult skeletal muscle associated MHC isoforms, the multiple exonic alignments (as previously described) from MyH1, MyH2 and MyH4 were obtained from the genomic sequence, and the newly annotated putative sequence data subjected to 5' and 3' RACE using primers designed to amplify all three fast isoforms (Table 1). RNA derived from three different muscles was used in the 5' and 3' RACE protocol to enrich for the various MHC gene transcripts since *Extensor digitorum longus *is known to predominantly express MyH1 and MyH4; *Soleus *MyH2 and MyH7 whilst *Quadriceps *is reported to express all four isoforms based on data from previous histochemical studies [19].

**Figure 2:**
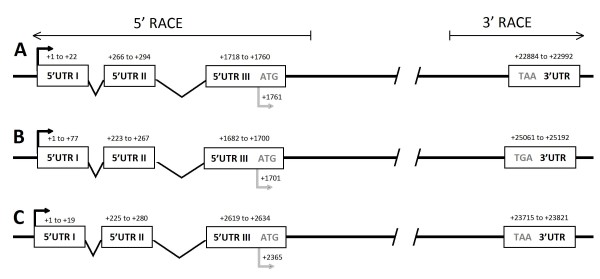
**Illustration of the relative 5' and 3' genomic structure of guinea pig MyH 1 (A), 2 (B) and 4 (C) derived from 5' and 3' RACE products indicating the transcription start site and exon positions**. Open boxes - individual exons; Thick black arrow - transcription start site; Thick grey arrow - translation start site; Numbers indicate the position relative to the transcription start site in the respective gene.

Both 5' and 3' RACE protocols resulted in the formation of specific race products which were subsequently cloned and sequenced. For each MHC transcript, 3' RACE sequences encoded approximately 400 bp of coding sequence followed by the 3' UTR, whilst 5' RACE sequences encoded approximately 300 bp of coding sequence followed by the 5' UTR. The 3' and 5' untranslated regions were used to identify the start and end of transcription for MyH 1, 2, and 4 (Figure 2). For all three genes or RACE products assessed, the resulting novel 5' UTRs encoded three distinct exons, the first two of which fell within a 100-200 bp region, the third was located more distantly and encoded the signal to instigate translation (ATG in this instance). The novel 3' UTRs encoded a single exon starting immediately downstream of the stop codon (**TAA **in MyH1 and MyH4, and **TGA **in MyH2) (Figure 2). All novel 3' and 5' sequences were deposited in GenBank (see Table 2 for accession numbers)

### Design of Specific Oligonucleotide Primers

**Figure 3:**
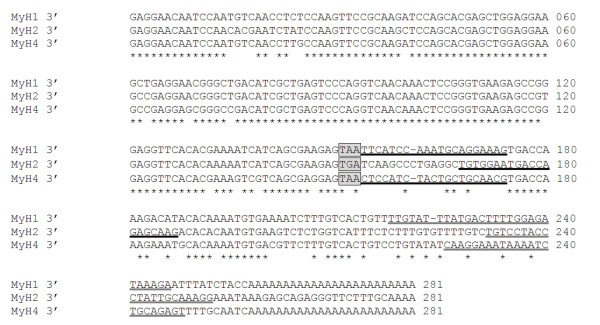
**Multiple sequence alignment of the 3' untranslated region of MyH 1, 2 and 4 respectively demonstrating isoform specificity in the regions to which quantitative PCR oligonucleotide primers were designed**. Grey boxes - translation termination signal; Black underline - forward primer; Grey underline - reverse primer; Star - nucleotide conserved between all three sequences.

**Figure 4:**
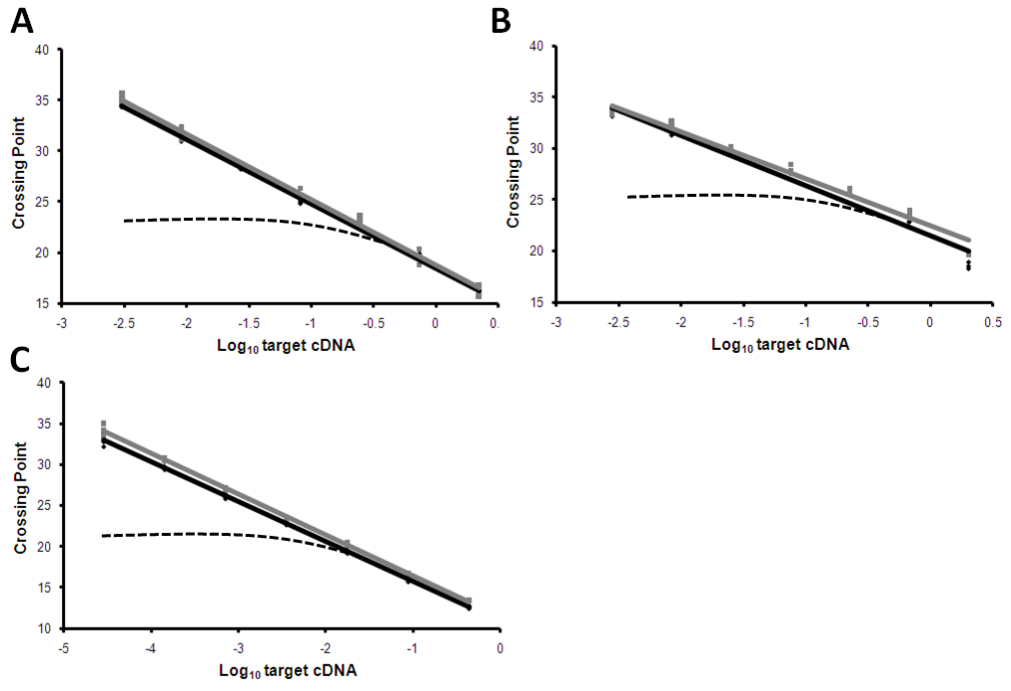
**Specificity of quantitative PCR reactions for each target MHC gene transcript**. Linearised vectors containing the 3' UTRs pertaining to MyH1 (A), 2 (B) and 4 (C) were adjusted to 2 ng/μl and serially diluted. Five-fold serial dilutions were then mixed 1:1 (vol/vol) with molecular biology grade water (control) or a combination of the two competing vectors at a concentration of 0.4 ng/μl and subjected to quantitative PCR with the respective MyH primer sets in triplicate. No cross-reaction was noted between any of the MyH primer sets and competing vectors. Black line - control reaction; Grey line - reaction with competitor vectors added; Axes - Log _10 _vector concentration against crossing point (Cp); Dashed line - predicted observation should cross-reaction occur.

Primers for MyH7β encoded mRNA were designed at the 3' of the coding sequence as determined by the full length MyH7β cDNA ENSCPOG00000004208 (Ensemble release 56). Due to the high degree of conservation between the remaining fast variant mRNAs, oligonucleotide primers were designed in the newly described 3' UTR where more divergence was noted, as determined by 3' RACE (Figure 3). Due to this high degree of homology, the fast MHC mRNA primers sets (MyH13'UTR, MyH23'UTR and MyH43'UTR) were subjected to a further assessment of specificity. Vectors containing the 3' UTRs pertaining to MyH1, 2 and 4 were linearised by *Bgl*1 endonuclease digestion (NEB), adjusted to 2 ng/μl and serially diluted. Five-fold serial dilutions were then mixed 1:1 (vol/vol) with molecular biology grade water (control) or a combination of the two competing vectors at a concentration of 0.4 ng/μl. Following quantitative PCR using the SYBR-Green detection system, no cross-reaction was observed between any of the primer pairs tested (Figure 4).

### Assessment of Three Distinct Guinea Pig Skeletal Muscles by Quantitative PCR

**Figure 5:**
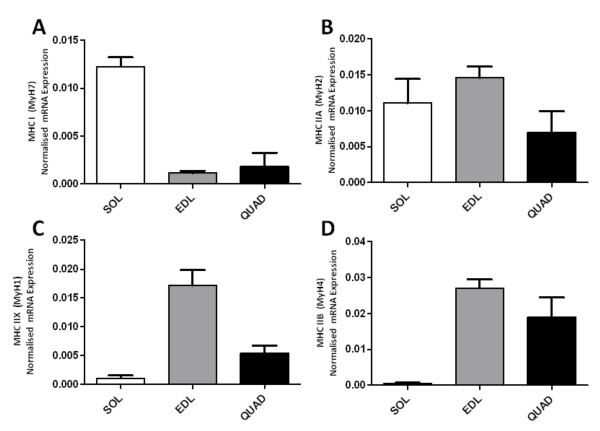
**Expression of MyH7 (A), MyH2 (B), MyH1 (C) and MyH4 (D) transcripts in three distinct skeletal muscles as determined by quantitative PCR reactions in triplicate normalised to total first-strand cDNA concentration**. White bar - *Soleus*; Grey bar - *Extensor digitorum longus*; Black bar - *Quadriceps*. Error bars indicate mean normalised expression + S.E.M; n = 6.

In order to validate further the utility of the oligonucleotide primers designed, three distinct guinea pig muscles were analysed in terms of their MHC mRNA expression. Based on histochemical studies of numerous laboratory species, including the guinea pig, *Soleus *is known to be a slow, postural muscle with a predominance of MHC I and MHC IIA muscle fibres and therefore by inference MyH7 and MyH2 mRNA variants. In contrast, *Extensor digitorum longus *has a faster contractile phenotype and a predominance of MHC IIX and MHC IIB muscle fibres (encoded by MyH1 and MyH4 mRNAs). Finally, *Quadriceps femoris *is known to express all muscle fibre types and therefore all variants of myosin heavy chain mRNA [19]. Quantitative PCR analysis of guinea samples from the above muscles revealed expression patterns as expected. MyH7 and MyH2 were most abundantly detected in the slow, postural *Soleus. *MyH1 and MyH4 were detected most abundantly in the fast twitch *Extensor digitorum longus*, whilst all four transcripts were detected at intermediate levels in the *Quadriceps *muscle (Figure 5)

## Conclusions

In this study, we partially characterised the structure of the myosin heavy chain multigene family in the common guinea pig (*Cavia porcellus*). As with other species studied, the MHC genes were found to be located in a head-to-tail fashion in the order MyH3/MyH2, MyH1/, MyH 4 and MyH8/MyH13. Although exact chromosomal locations are still unknown due to restricted genomic annotation, we found the MyH7β gene to be located in a distinct contig, consistent with its location on a distal chromosome in other species. We have designed and tested specific oligonucleotide primers for MyH7β, MyH2, MyH1 and MyH4 mRNA variants, and for the first time, are able to successfully differentiate and quantify the adult skeletal muscle associated MHC variants by quantitative PCR analysis.

The methods developed here are suitable for the characterisation of muscle myosin heavy chain complement at the molecular level from animal tissue samples and biopsy material. Furthermore, the publication of these specific oligonucleotide primers for the guinea pig myosin heavy chain variants will enable researchers to rapidly and accurately quantify acute changes in MHC expression in either developmental or in guinea pig disease models where a marker of altered skeletal muscle function is required.

## Methods

### Extraction of Total RNA

*Extensor digitorum longus, soleus *and *quadriceps femoris *were carefully dissected from 3 month old Dunkin Hartley strain guinea pigs (Harlan, UK) (n = 6) and immediately snap frozen in liquid nitrogen. Total RNA was extracted from 100 mg of sample using TRIzol regent (Invitrogen) according to standard procedure. Contaminating genomic DNA was removed by DNase digestion (Promega) as specified by the manufacturers. The resulting total RNA was resuspended in molecular biology grade water (Promega). All RNA was stored at -80°C prior to use.

### Reverse Transcription

First strand complementary DNA (cDNA) was reverse transcribed from 1 μg total RNA using random hexamers and Moloney murine leukemia virus reverse transcriptase (MMLV) in a final volume of 25-μl as described by the manufacturer (Promega).

### Primer Design

All primers were designed using Primer3 software and sourced from MWG Eurofins Operon. To enforce specificity, all base mis-matches were directed towards the 5' end of each oligonucleotide primer.

### Polymerase Chain Reaction (PCR)

End-point PCR reactions were performed on 5 μl cDNA in AmpliTaq Gold Buffer (ABI), 1.5 mM MgCl _2_, 0.2 mM dNTP mix (Promega), 0.25 mM forward and reverse oligonucleotide primers (Eurofins) and 1.25 U AmpliTaq (ABI) in a total reaction volume of 50 μl. Cycling parameters were 94°C for 10 minutes; 35 cycles of 94°C for 30 s, 60°C for 30 s, 72°C for 30 s followed by a final extension at 72°C for 10 minutes. PCR products were resolved on 1.2% (w/v) agarose gel (Melford), stained for 30 minutes in ethidium bromide (0.5 μg/mL) and visualised under a UV light source.

Quantitative PCR reactions were performed in triplicate on 5 μl cDNA in SYBR 1 Master mix (Roche), 0.25 mM forward and reverse primers in a final volume of 15 μl. Cycling parameters were 95°C for 5 minutes prior to; 35 cycles of 10 seconds 95°C, 10 seconds 55°C and 30 seconds at 72°C. Single signal acquisition was set to read at 72°C. All reactions were run on a 384-well microplate on a LightCycler LC480 (Roche) configured for SYBR green determination as specified by the manufacturers. Melt curve analysis was performed at the end of each completed analysis run to ensure only the specific product was amplified. All quantitative PCR data were normalised to the total first strand cDNA concentration following reverse transcription using OliGreen (Invitrogen). OliGreen determination has been previously validated as an alternative to the use of traditional housekeeping genes [20,21].

### Cloning

Where indicated, PCR products were cloned using the TOPO TA cloning system (Invitrogen) according to the manufacturer's standard protocol. Inserts were confirmed by sequencing which was bidirectional, and undertaken by GeneService (Nottingham) using primers in the vector sequences.

### RNA Ligase-Mediated Rapid Amplification of 5' and 3' cDNA Ends (Race)

RACE was carried out on 3000 ng of total RNA using the GeneRacer Kit (Invitrogen). All procedures were carried out in accordance with the manufacturer's instructions. 5'RACE nested PCR reactions were carried out using primer Reverse_GSP_2 directed against exon 2 (5'-CAGGCTGCGTAACGCTCTTTGAGGTTGT-3') for the first round and primer Reverse_Nested_GSP_2 located 36 bp upstream of primer Reverse_GSP_2 (5'-CTCGTGCAGGTGGGTCATCATGG-3'), for the second round. 3'RACE nested PCR reactions were carried out using primer Forward_GSP_2 directed against exon 36 (5'-CATGATGGCCGAGGAGCTGAAGAAG-3') for the first round and primer Forward_Nested_GSP_2 located 105 bp downstream of primer Reverse_GSP_2 (5'-AAGGGCGGCAAGAAGCAGATCCAGAAG-3'), for the second round. PCR generated products were cloned using the TOPO TA cloning kit for sequencing (Invitrogen) and sequenced (Geneservice).

### Sequence Alignment and Bioinformatics

Due to the lack of sequence annotation within the *Cavia porcellus *genome, likely conserved regions were located by obtaining cDNA sequences pertaining to MyH1, MyH2 and MyH4 mRNA variants (encoding MHC IIX, IIA and IIB respectively) from mouse (*Mus musculus*) and rat (*Rattus norvegicus*) via Ensemble (release 56). All sequences were aligned using the ClustalW2 application (EBI) configured for DNA analysis.

## Competing interests

The authors declare that they have no competing interests.

## Authors' contributions

DPT completed the bioinformatics, molecular biology and prepared the original manuscript.

DPT, SWJ, RGB and TP were involved in the interpretation of data and strategic planning.

All authors read and approved the final manuscript.

## References

[B1] Rinaldi C Haddad F Bodell PW Qin AX Jiang WH Baldwin KM Intergenic bidirectional promoter and cooperative regulation of the IIx and IIb MHC genes in fast skeletal muscle American Journal of Physiology-Regulatory Integrative and Comparative Physiology 2008 295 R208 R218 10.1152/ajpregu.00134.2008 PMC2494810 18434443

[B2] Weiss A Leinwand LA The mammalian myosin heavy chain gene family Annual Review of Cell and Developmental Biology 1996 12 417 439 10.1146/annurev.cellbio.12.1.417 8970733

[B3] Rossi AC Mammucari C Argentini C Reggiani C Schiaffino S Two novel/ancient myosins in mammalian skeletal muscles: MYH14/7b and MYH15 are expressed in extraocular muscles and muscle spindles The Journal of Physiology 2010 588 353 364 10.1113/jphysiol.2009.181008 19948655 PMC2821527

[B4] Mascarello F Patruno M Toniolo L Reggiani C Maccatrozzo L Phenotypic expression of 2b myosin heavy chain isoform: a comparative study among species and different muscles Veterinary Research Communications 2009 33 105 107 10.1007/s11259-009-9301-9 19578947

[B5] Harridge SDR Plasticity of human skeletal muscle: gene expression to in vivo function Experimental Physiology 2007 92 783 797 10.1113/expphysiol.2006.036525 17631518

[B6] Mascarello F Maccatrozzo L Patruno M Toniolo L Reggiani C 2B Myosin Heavy Chain Isoform Expression in Bovine Skeletal Muscle Veterinary Research Communications 2004 28 201 204 10.1023/B:VERC.0000045406.59936.20 15372957

[B7] Pette D Staron RS Transitions of muscle fiber phenotypic profiles Histochemistry and Cell Biology 2001 115 359 372 11449884 10.1007/s004180100268

[B8] Fink B Egl M Singer J Fuerst M Bubenheim M Neuen-Jacob E Morphologic changes. in the vastus medialis muscle in patients with osteoarthritis of the knee Arthritis and Rheumatism 2007 56 3626 3633 10.1002/art.22960 17968889

[B9] Nakao K Minobe WA Roden RL Bristow MR Leinwand LA Myosin heavy chain gene expression in human heart failure Circulation 1997 96 I117 I118 10.1172/JCI119776 PMC508434 9410916

[B10] Ottenheijm CAC Heunks LMA Dekhuijzen RPN Diaphragm adaptations in patients with COPD Respiratory Research 2008 9 10.1186/1465-9921-9-12 18218129 PMC2248576

[B11] Laviolle B Pape D Verdier MC Lavenu A Bellissant E Hemodynamic and histomorphometric characteristics of heart failure induced by aortic stenosis in the guinea pig: comparison of two constriction sizes Canadian Journal of Physiology and Pharmacology 2009 87 908 914 10.1139/Y09-083 19935898

[B12] Bowyer J Heapy CG Flannelly JK Waterton JC Maciewicz RA Evaluation of a magnetic resonance biomarker of osteoarthritis disease progression: doxycycline slows tibial cartilage loss in the Dunkin Hartley guinea pig International Journal of Experimental Pathology 2009 90 174 181 10.1111/j.1365-2613.2008.00634.x 19335556 PMC2676705

[B13] Canning BJ Chou YL Using guinea pigs in studies relevant to asthma and COPD Pulmonary Pharmacology & Therapeutics 2008 21 702 720 10.1016/j.pupt.2008.01.004 18462968 PMC2882537

[B14] Brooke M Kaiser K 3 myosin adenosine triphosphatase systems - nature of their pH lability and sulfhydryl dependence Journal of HIstochemistry & Cytochemistry 1970 18 670 10.1177/18.9.670 4249441

[B15] Mizunoya W Wakamatsu J Tatsumi R Ikeuchi Y Protocol for high-resolution separation of rodent myosin heavy chain isoforms in a mini-gel electrophoresis system Analytical Biochemistry 2008 377 111 113 10.1016/j.ab.2008.02.021 18358820

[B16] Tonge DP Jones SW Parr T Bardsley R Doherty M Maciewicz RA β2-Adrenergic agonist-induced hypertrophy of the quadriceps skeletal muscle does not modulate disease severity in the rodent meniscectomy model of osteoarthritis Osteoarthritis and Cartilage 2009 18 555 562 10.1016/j.joca.2009.11.014 20060953 PMC2849930

[B17] Andersen JL Schiaffino S Mismatch between myosin heavy chain mRNA and protein distribution in human skeletal muscle fibers American Journal of Physiology-Cell Physiology 1997 272 C1881 C1889 10.1152/ajpcell.1997.272.6.C1881 9227417

[B18] Gustafson TA Markham BE Morkin E Effects of Thyroid-Hormone on Alpha-Actin and Myosin Heavy Chain Gene-Expression in Cardiac and Skeletal-Muscles of the Rat - Measurement of Messenger-RNA Content using Synthetic Oligonucleotide Probes Circulation Research 1986 59 194 201 3742743 10.1161/01.res.59.2.194

[B19] Ariano MA Edgerton VR Armstrong RB Hindlimb muscle fibre population of five mammals J. Histochem. Cytochem 1973 21 51 55 4348494 10.1177/21.1.51

[B20] Rhinn H Marchand-Leroux C Croci N Plotkine M Scherman D Escriou V Housekeeping while brain's storming Validation of normalizing factors for gene expression studies in a murine model of traumatic brain injury BMC Molecular Biology 2008 9 62 10.1186/1471-2199-9-62 18611280 PMC2500043

[B21] Rhinn H Scherman D Escriou V One-step quantification of single-stranded DNA in the presence of RNA using Oligreen in a real-time polymerase chain reaction thermocycler Anal Biochem 2008 372 116 118 10.1016/j.ab.2007.08.023 17963709

